# The diagnostic performance and clinical value of deep learning-based nodule detection system concerning influence of location of pulmonary nodule

**DOI:** 10.1186/s13244-023-01497-4

**Published:** 2023-09-19

**Authors:** Seulgi You, Ji Hyun Park, Bumhee Park, Han-Bit Shin, Taeyang Ha, Jae Sung Yun, Kyoung Joo Park, Yongjun Jung, You Na Kim, Minji Kim, Joo Sung Sun

**Affiliations:** 1 Department of Radiology, Ajou University School of Medicine, Suwon, Republic of Korea; 2 Office of Biostatistics, Ajou Research Institute for Innovative Medicine, Ajou University Medical Center, Suwon, Republic of Korea; 3 Departments of Biomedical Informatics, Ajou Research Institute for Innovative Medicine, Ajou University School of Medicine, Suwon, Republic of Korea

**Keywords:** Deep learning, Chest radiography, Solitary pulmonary nodule

## Abstract

**Background:**

The deep learning-based nodule detection (DLD) system improves nodule detection performance of observers on chest radiographs (CXRs). However, its performance in different pulmonary nodule (PN) locations remains unknown.

**Methods:**

We divided the CXR intrathoracic region into non-danger zone (NDZ) and danger zone (DZ). The DZ included the lung apices, paramediastinal areas, and retrodiaphragmatic areas, where nodules could be missed. We used a dataset of 300 CXRs (100 normal and 200 abnormal images with 216 PNs [107 NDZ and 109 DZ nodules]). Eight observers (two thoracic radiologists [TRs], two non-thoracic radiologists [NTRs], and four radiology residents [RRs]) interpreted each radiograph with and without the DLD system. The metric of lesion localization fraction (LLF; the number of correctly localized lesions divided by the total number of true lesions) was used to evaluate the diagnostic performance according to the nodule location.

**Results:**

The DLD system demonstrated a lower LLF for the detection of DZ nodules (64.2) than that of NDZ nodules (83.2, *p *= 0.008). For DZ nodule detection, the LLF of the DLD system (64.2) was lower than that of TRs (81.7, *p < *0.001), which was comparable to that of NTRs (56.4, *p *= 0.531) and RRs (56.7, *p *= 0.459). Nonetheless, the LLF of RRs significantly improved from 56.7 to 65.6 using the DLD system (*p *= 0.021) for DZ nodule detection.

**Conclusion:**

The performance of the DLD system was lower in the detection of DZ nodules compared to that of NDZ nodules. Nonetheless, RR performance in detecting DZ nodules improved upon using the DLD system.

**Critical relevance statement:**

Despite the deep learning-based nodule detection system’s limitations in detecting danger zone nodules, it proves beneficial for less-experienced observers by providing valuable assistance in identifying these nodules, thereby advancing nodule detection in clinical practice.

**Key points:**

• The deep learning-based nodule detection (DLD) system can improve the diagnostic performance of observers in nodule detection.

• The DLD system shows poor diagnostic performance in detecting danger zone nodules.

• For less-experienced observers, the DLD system is helpful in detecting danger zone nodules.

**Graphical Abstract:**

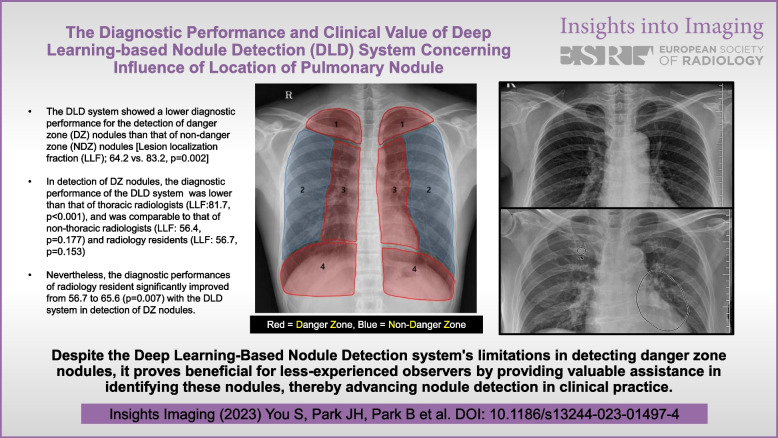

**Supplementary Information:**

The online version contains supplementary material available at 10.1186/s13244-023-01497-4.

## Background

Chest radiographs (CXRs) are the first-line diagnostic imaging tests for the detection of lung nodules [1]. However, their sensitivity in detecting lung nodules varies from 44 to 87% [1–4]. CXRs demonstrate frequent reading errors, and the improvement over the past decades has been insufficient [5].

Many variables affect the ability to detect pulmonary nodules (PNs) on CXR; the major contributing factors are PN location (e.g., poorly penetrated area of the lung) and anatomical background (e.g., superimposed structures and surrounding complexity). Therefore, PNs located in the apical lung as well as in the paramediastinal and retrodiaphragmatic areas are more likely to be missed by observers. Interestingly, several studies have demonstrated that missed lung cancers are commonly located in these areas [6–10].

Recently, deep learning-based nodule detection (DLD) systems, which have been developed to assist in nodule detection, have shown promising results. DLD systems improve observer sensitivity and reduce the number of false-positive findings [2, 11, 12]. Furthermore, one of the expected roles of the DLD system is to assist observers in detecting PNs in hard-to-detect areas (apical, paramediastinal, and retrodiaphragmatic areas). However, the usefulness of the DLD system in aiding in the detection of PNs in these areas has not yet been evaluated.

This study aimed to investigate the diagnostic performance and clinical value of the DLD system according to the location of PNs.

## Materials and methods

This retrospective study was approved by the Institutional Review Board of our institution, and the requirement for informed consent was waived.

### Location of PNs

**Fig. 1:**
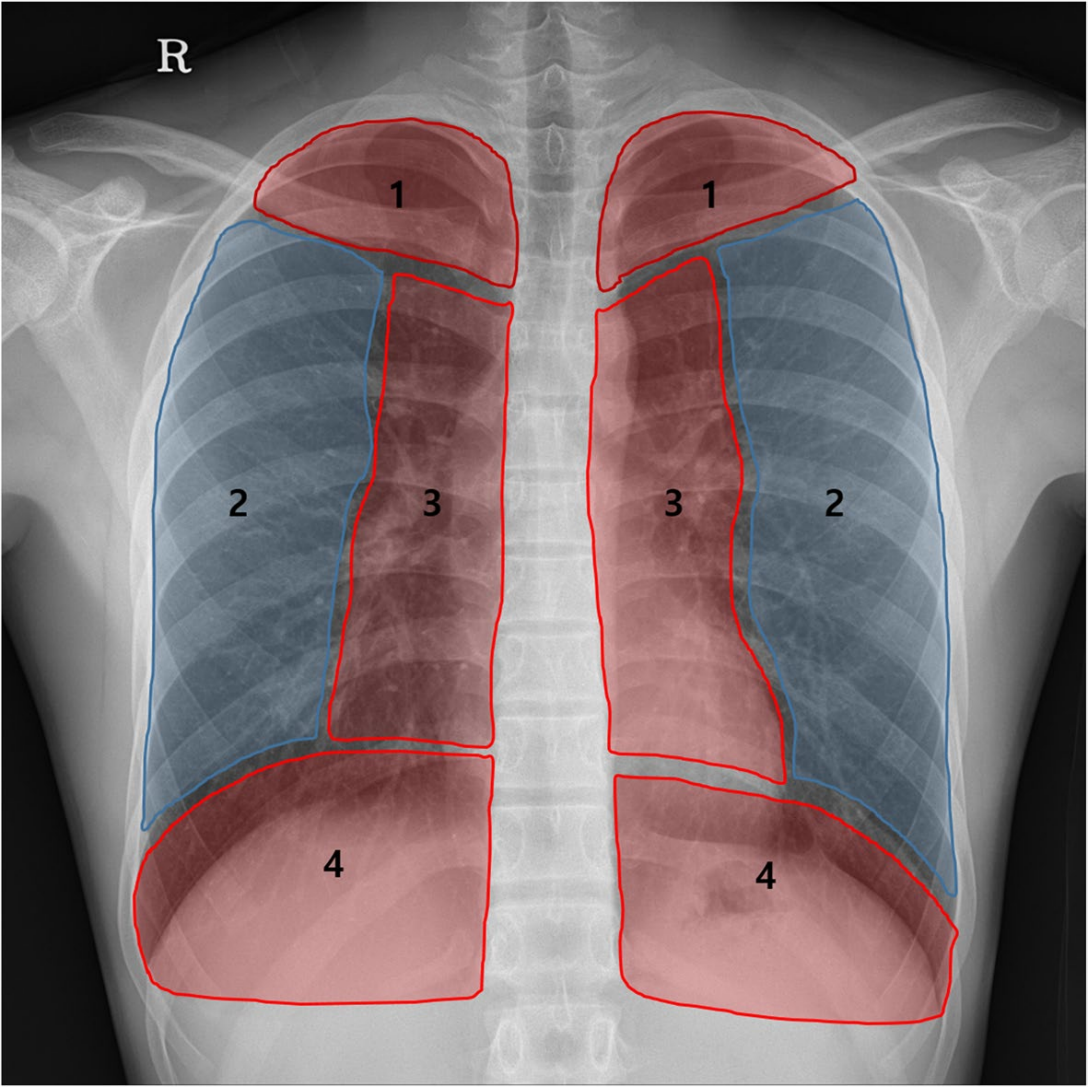
Schematic diagram of the danger and non-danger zones. The lung field on the chest radiograph is divided into four regions: (1) apical, (2) lateral pulmonary, (3) paramediastinal, and (4) retrodiaphragmatic regions. Danger zone: regions 1, 3, and 4 vs. non-danger zone: region 2

We analyzed the detection performance of DLD according to PN location. Therefore, we adopted the concept of danger (DZ) and non-danger zones (NDZ) in the lungs. The lung field on the CXR was divided into four regions based on the anatomy as follows: region 1 (apical), above the inferior margin of the clavicles; region 2 (lateral pulmonary), mid to outer region of the lung field; region 3 (paramediastinal), between the apical and retrodiaphragmatic regions, medial aspect of 3 cm lateral to the paravertebral line, including the pulmonary hila and cardiac shadow; region 4 (retrodiaphragmatic), inferior to 1 cm above the diaphragmatic line (Fig. 1). These regions were further classified into two groups based on the anatomical location influencing the detection of PNs: DZ and NDZ. Regions 1, 3, and 4 were designated as DZ, while region 2 was designated as NDZ [13].

### Preparation of the dataset

**Fig. 2:**
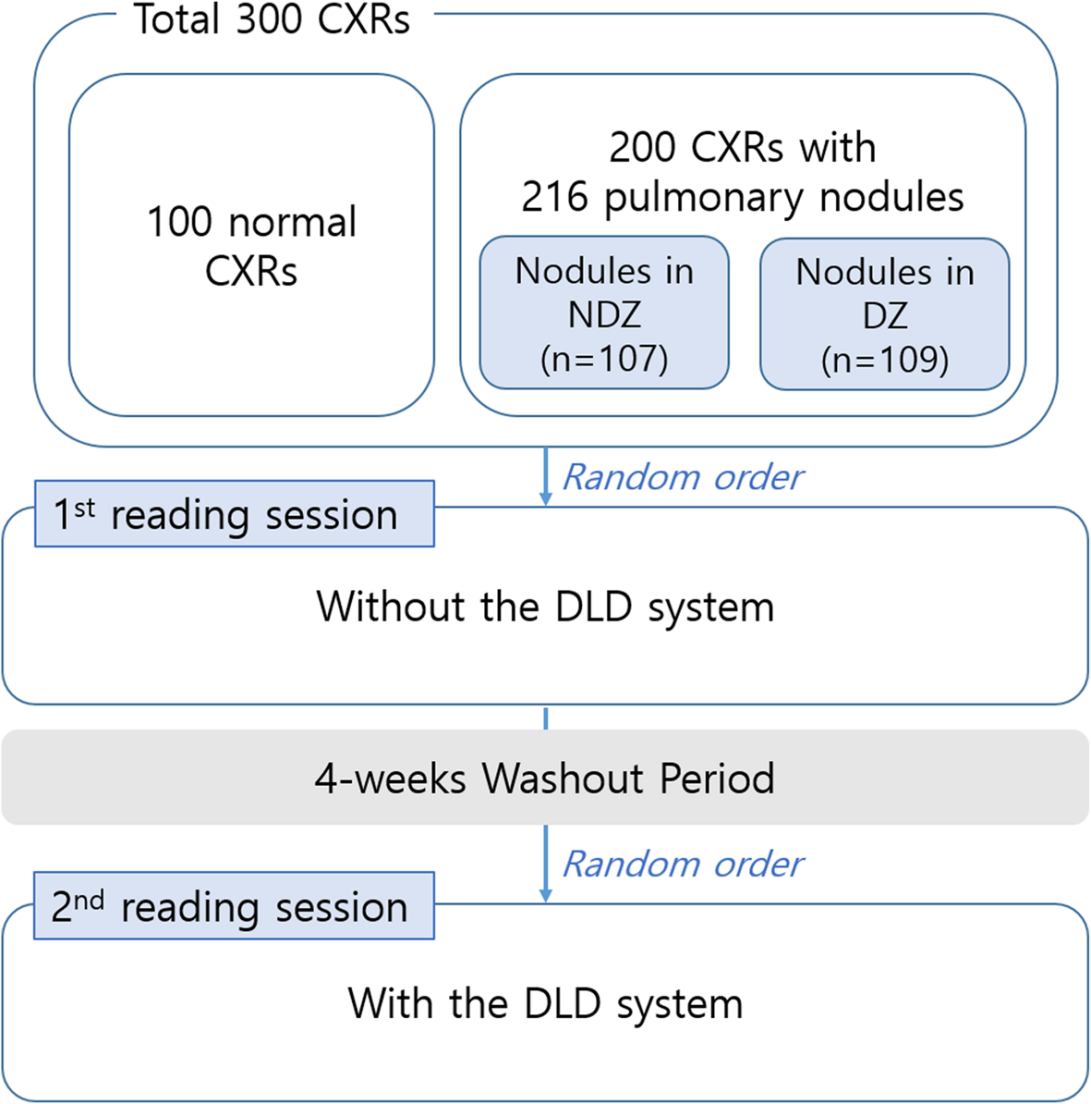
Flowchart of the performance test. The dataset included 100 normal chest radiographs (CXRs) and 200 CXRs with 216 pulmonary nodules. The 216 pulmonary nodules consisted of 107 nodules in the non-danger zone (NDZ) and 109 nodules in the danger zone (DZ). Eight observers reviewed each chest radiograph twice, with and without the deep learning-based nodule detection (DLD) system. The order of each radiograph was randomly assigned before each session and a 4-week washout period was ensured between two reading sessions

In this retrospective study, we used CXRs of adults (age ≥18 years), which were obtained between January 2017 and December 2020 at a single tertiary hospital. A total of 300 CXRs were prepared for analysis, including 100 normal CXRs and 200 CXRs with PNs (Fig. 2). All the CXRs in the dataset were taken in the posteroanterior position using one of 5 scanners (Table S1). Typical imaging parameters at our institution for CXRs were tube voltage of 120 kVp, 500 mA with automatic exposure, control speed of 125, and source-to-image distance of 180 cm. The detector had an image size of 41×41cm field of view. All 300 images of the CXR dataset were anonymized using a commercially available software (AVIEW, Coreline, Seoul, South Korea).

#### Dataset of normal CXRs

For normal CXR, two thoracic radiologists (TRs) performed the selection process. We included all the CXRs obtained between January 2017 and December 2017 in this group that met the following inclusion criteria: (1) patients who underwent chest radiography and chest computed tomography (CT) within a 4-week interval, (2) no identified PN or mass in both imaging modalities, (3) no combined significant parenchymal abnormality, and (4) adequate image quality for nodule detection as judged by thoracic radiologists. A total of 2817 normal CXRs were identified. We randomly selected 100 CXRs from the normal CXR dataset.

#### Dataset of CXRs with PNs

We collected CXRs with PNs meeting the following inclusion criteria: (1) patients in whom PNs were detected on chest CT and were also identified on CXR, (2) time interval between chest CT and CXR within 4 weeks, (3) mean diameter of PNs on chest CT of > 5 mm and < 30 mm, (4) no internal calcification of the PNs, (5) no combined significant pulmonary abnormality interfering with PN detection, and (6) adequate image quality for nodule detection as judged by thoracic radiologists. First, 878 CXRs with 1022 PNs were identified. Thereafter, we randomly selected 200 CXRs with 216 PNs, maintaining a 1:1 ratio of NDZ to DZ nodules.

#### Reference standard establishment

A radiologist with 4 years of experience in thoracic radiology reviewed all 878 CXRs with PNs, chest CT images, and clinical data. The TR labeled and annotated the PNs on CXR, and classified their location (NDZ and DZ). The nodule visibility score was graded on a 4-point scale, where 1, 2, 3, and 4 points correspond to very subtle, subtle, moderately visible, and clearly visible, respectively. On the chest CT of the lung window setting, the TR measured the mean diameter of the PNs and determined the nodule solidity (solid vs. subsolid). All the malignant lesions were confirmed via surgical resection or biopsy. Benign lesions were confirmed by biopsy or were clinically established (stable lesion for at least 2 years or improvement with conservative treatment).

### Information regarding the DLD system

We used a commercially available DLD system (Med-Chest X-ray system, version 1.0.1, VUNO, Seoul, South Korea) for PN detection, which has been commercially available in South Korea and Europe since July 2019 and June 2020, respectively. This system was developed by training a multi-task convolutional neural network with 15,609 CXRs [14].

### Diagnostic performance of DLD system for nodule detection

All 300 anonymized CXRs were uploaded to the DLD system server to evaluate the diagnostic performance of PN detection. The DLD system reported the result with the percentage of possibility and marked the location of the PN on CXR with the masked layer.

### Diagnostic performance of observers for nodule detection

Eight observers (two board-certified TRs, two board-certified non-thoracic radiologists [NTRs], and four radiology residents [RRs]) participated as observers. Observer performance was stratified into the TR, NTR, and RR groups. All 300 anonymized CXRs were uploaded into a custom folder on a picture archiving and communication systems (PACS) server (G3, Infinitt Healthcare, Seoul, South Korea). The observers evaluated CXRs using PACS, which was familiar to them since it was used daily in actual clinical practice. They did not have access to any other information or related imaging findings (e.g., chest CT images). They evaluated the images without information concerning the reference standard, final diagnosis, ratio of the number of CXRs containing PNs to the number of normal CXRs, and number of nodules per CXR. They were instructed to focus on the PNs and ignore the other findings. They were allowed to zoom in or control the CXR window settings. The observers were requested to mark the location of the PNs on the CXRs and, subsequently rate their confidence level based on a 5-point scale. Scores of 1 and 5 points represented a low and a high likelihood of it being a nodule, respectively. The observers referred to reference images corresponding to scores of 1–5 points. The observers performed two sessions of CXR readings. In the first session, they evaluated all the CXRs without the DLD system results. After 4 weeks, they evaluated the chest radiographs again with the DLD system results. To reduce bias, the order of the radiographs was randomly assigned in each session (Fig. 2).

### Statistical analysis

The overall diagnostic performance of PN detection was evaluated using the figure of merit from the jackknife alternative free-response receiver operating characteristic (JAFROC) analysis (RJafroc software, version 1.2.0) [15]. False-positive findings per image (FPPI) were calculated as follows: the number of false-positive markings divided by the total number of radiographs in the dataset [12]. All observer markings with all confidence scores were included in the FPPI calculations. The FPPI was compared using the McNemar test. Diagnostic performance according to the location of PNs was evaluated using the metric of lesion localization fraction (LLF). LLF was calculated as the number of correctly localized lesions divided by the total number of true lesions. All the observer markings with all confidence scores were included in the LLF calculations. LLFs of the DLD systems and observers were compared using the generalized estimating equation. Post hoc analysis was performed by Tukey’s Honest Significant Difference test. A *p*-value of < 0.05 indicated statistical significance. To correct for multiple testing, the Bonferroni correction of *p*-value was applied. Statistical analyses were performed using R software (version 3.6.1; The R Project for Statistical Computing, Vienna, Austria) and MedCalc (version 20.010; MedCalc Software Ltd., Ostend, Belgium).

## Results

### Patient information and characteristics of the PNs

A total of 300 CXRs from 100 patients (mean age, 46.5 ± 14.7 years; age range, 18–75 years; sex, 50 men and 50 women) with normal CXRs and 200 patients (mean age, 60.0 ± 13.2 years; age range, 18–88 years; sex, 114 men and 86 women) with CXRs containing PNs were included. The CXRs with the PN dataset included 216 PNs (107 NDZ and 109 DZ nodules). One PN was included in 188 CXRs; eight and four CXRs contained two and three PNs per image, respectively. The mean size of the PNs in DZ was significantly larger than that in NDZ (18.12 and 15.81 mm, respectively, *p *= 0.005). A summary of the PN characteristics is presented in Table 1.

**Table 1:** Patient information and characteristics of pulmonary nodules

**Patient information**				
	**Patients without nodule**		**Patients with nodules**	
No. of men	50		114	
No. of women	50		86	
Mean age	46.51 ± 14.66		60.09 ± 13.23	
**Number of nodules per chest radiograph**				
One nodule	188			
Two nodules	8			
Three nodules	4			
**Characteristics of pulmonary nodules**				
	**Non-danger zone nodules (*n *= 107)**	**Danger zone nodules (*n *= 109)**		***p*-value**
Size (cm)	15.81 ± 6.22	18.12 ± 5.61		0.005*
≤ 2.0	80	68		
2.1 - 3.0	27	41		
Nodule solidity				0.148
Solid	89	98		
Subsolid	18	11		
Part-solid	15	11		
Nonsolid	3	0		
Disease entity				0.700
Malignant	67	71		
Benign	40	38		
Lobar distribution				0.088
Right upper	29	29		
Right middle	10	3		
Right lower	19	28		
Left upper	29	21		
Left lower	20	28		
Visibility score				< 0.001*
1	6	6		
2	22	24		
3	31	59		
4	48	20		
Location of danger zone nodules				
Apical lung zone		33		
Paramediastinal		59		
Retrodiaphragmatic		17		

^*^p < 0.05 was regarded as statistically significant

### Overall diagnostic performance of the DLD system and the observers

Table 2 shows the JAFROC figure of merit of the DLD system and the observers with and without the use of the DLD system. In the first session (without the DLD system), the overall diagnostic performance of NTRs (0.850) and RRs (0.806) in detecting PNs was similar to that of the DLD system (0.836). However, the diagnostic performance of TRs (0.895) was better than that of the DLD system (*p *= 0.006). In the second session (with the DLD system), the diagnostic performances of TRs (0.932) and RRs (0.862) were significantly higher than those in the first session. The diagnostic performance of NTRs did not change significantly.

**Table 2:** JAFROC figure of merit and FPPI of the DLD system and observers

	JAFROC figure of merit				FPPI		
	Session 1	*p*-value (vs. DLD)	Session 2	*p-*value (vs. session 1)	Session 1	Session 2	*p-*value
DLD system	0.836				0.20		
Thoracic radiologists	0.895	0.006*	0.932	< 0.001*	0.17 (104/600)	0.12 (73/600)	< 0.001*
Non-thoracic radiologists	0.850	0.777	0.860	> 0.999	0.12 (72/600)	0.08 (47/600)	< 0.001*
Radiology residents	0.806	0.204	0.862	0.009*	0.19 (232/1200)	0.12 (140/1200)	< 0.001*

*DLD* Deep learning-based nodule detection, *FPPI* False-positive findings per image, *JAFROC* Jackknife alternative free-response receiver operating characteristic

^*^p < 0.05 was regarded as statistically significant. For group-averaged comparison, corrected *p-*values are presented (multiplied by 3)

The FPPI of the DLD system, TRs, NTRs, and RRs were 0.20, 0.17, 0.12, and 0.19, respectively. The FPPI decreased significantly in all the observer groups using the DLD system (Table 2).

Considering further details concerning false-negative lesions, the DLD system demonstrated better performance in the detection of PNs in the NDZ than in the DZ; therefore, false-negative nodules missed by the DLD system were more frequently identified in the DZ (39/109, 35.8%) than in the NDZ (18/107, 16.9%). Considering the location, of the 39 missed nodules in DZ, 28, 7, and 4 missed nodules were identified in the paramediastinal, retrodiaphragmatic, and apical areas, respectively. Considering the size, of the 39 missed nodules in DZ nodules, 35 missed nodules (89.7%) were < 2 cm in size and four missed nodules (11.4%) were > 2 cm. All missed nodules > 2 cm were identified in the paramediastinal area and all missed nodules located in the NDZ were < 2 cm.

### Diagnostic performance of the DLD system and the observers according to the nodule location

**Fig. 3:**
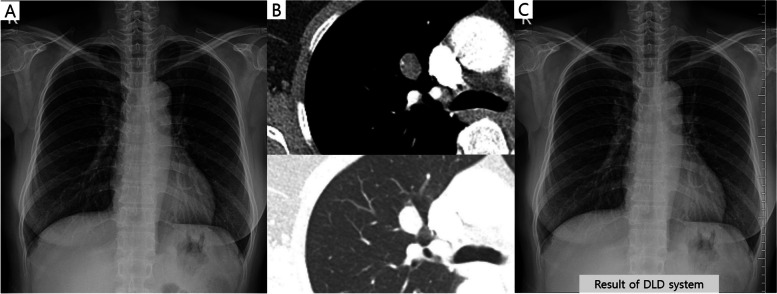
A case of a 53-year-old female patient with a pulmonary nodule. The nodule is located in the right paramediastinal area (danger zone) on the chest radiograph (**a**). Chest computed tomography revealed a 1.5-cm oval shape nodule in her right upper lobe (**b**). The deep learning-based nodule detection (DLD) system did not detect this nodule (**c**). In contrast, five out of the eight observers detected this nodule without the use of the DLD system

Table 3 shows the LLFs of the DLD system and the observers according to the nodule location. The diagnostic performance of the DLD system was better for the detection of PNs in the NDZ than in the DZ (Fig. 3). Similarly, the NTRs and RRs showed significantly better performance in the detection of PNs in the NDZ than in DZ. However, TRs showed similar diagnostic performance for the detection of PNs, regardless of the nodule location (Table 3).

**Table 3:** The LLF of the DLD system and the observers according to the nodule location

	Non-danger zone	Danger zone	*p*-value
DLD system	83.2 (89/107)	64.2 (70/109)	0.008*
Thoracic radiologists	84.6 (181/214)	81.7 (178/218)	> 0.999
Non-thoracic radiologists	77.1 (165/214)	56.4 (123/218)	< 0.001*
Radiology residents	75.5 (323/428)	56.7 (247/436)	< 0.001*

*DLD* Deep learning-based nodule detection, *LLF* Lesion localization fraction

^*^p < 0.05 was regarded as statistically significant. For group-averaged comparison, corrected *p-*values are presented (multiplied by 4). Denominator for the LLF calculation corresponds to the total number of true lesions multiplied by the number of readers in the respective group

The diagnostic performance of the DLD system according to the PN location was compared with that of the observers. In NDZ nodules, the LLF of the DLD system was comparable to that of TRs (83.2, 84.6, respectively; *p *= > 0.999). The LLF of the DLD system was slightly higher than those of the NTRs (77.1) and RRs (77.5). However, they were not statistically significant (*p *= 0.624 and *p *= 0.276, respectively). In the DZ nodules, the LLF of the DLD system (64.2) was significantly lower than that of the TRs (81.7, *p *< 0.001), which was slightly higher than that of the NTRs (56.4, *p *= 0.531) and RRs (56.7, *p *= 0.459).

### Subgroup analysis of the diagnostic performance of the DLD system and observers in the DZ nodules

Table 4 shows the subgroup analysis of the diagnostic performance of nodules in the DZ according to the location, visibility score, and size. When DZ was subdivided into three regions, the diagnostic performance of the DLD system was significantly higher in the apical area (87.9) than in the paramediastinal (52.5) and retrodiaphragmatic areas (58.8, *p *= 0.002). In the post hoc analysis, the DLD system showed significantly better diagnostic performance in detecting apical lung nodules than paramediastinal nodules (*p *= 0.004) (Table 5). A tendency towards higher the visibility score, higher the diagnostic performance was observed for the DLD system and all observer groups (Table 4). Post hoc analysis demonstrated better diagnostic performance of the DLD system in detecting nodules with high visibility (visibility score 4) than in nodules with subtle visibility (visibility score, 1 and 2 points; *p *= 0.011 and *p *= 0.034, respectively) (Table 5). In addition, the diagnostic performance was significantly higher when the nodule size was > 2 cm in the DLD system and all observer groups.

**Table 4:** Subgroup analysis of the LLFs in danger zone nodule

	DLD system	*p-*value	Thoracic radiologists	*p-*value	Non-thoracic radiologists	*p-*value	Radiology residents	*p-*value
Location								
Apical lung zone	87.9 (29/33)	0.002*	84.9 (56/66)	0.319	69.7 (46/66)	0.032*	65.9 (87/132)	0.120
Paramediastinal	52.5 (31/59)		78.8 (93/118)		53.4 (63/118)		57.6 (136/236)	
Retrodiaphragmatic	58.8 (10/17)		85.3 (29/34)		41.2 (14/34)		35.3 (24/68)	
Visibility score								
1	16.7 (1/6)	0.169	33.3 (4/12)	0.129	16.7 (2/12)	0.271	20.8 (5/24)	0.066
2	50.0 (12/24)		58.3 (28/48)		33.3 (16/48)		41.7 (40/96)	
3	64.4 (38/59)		90.7 (107/118)		60.2 (71/118)		58.5 (138/236)	
4	95.0 (19/20)		97.5 (39/40)		85.0 (34/40)		80.0 (64/80)	
Size								
≤ 2 cm	48.5 (33/68)	< 0.001*	74.3 (101/136)	< 0.001*	47.1 (64/136)	< 0.001*	47.8 (130/272)	< 0.001*
> 2 cm	90.2 (37/41)		93.9 (77/82)		71.9 (59/82)		71.3 (117/164)	

*DLD* Deep learning-based nodule detection, *LLF* Lesion localization fraction

^*^p < 0.05 was regarded as statistically significant. Denominator for the LLF calculation corresponds to the total number of true lesions multiplied by the number of readers in the respective group

**Table 5:** *p*-value of post hoc analysis for the comparison of LLFs in danger zone nodule

	Location of pulmonary nodule			Visibility score					
	Apical vs. paramediastinal	Apical vs. retrodiaphragmatic	Paramediastinal vs. retrodiaphragmatic	Score 1 vs. 2	Score 1 vs. 3	Score 1 vs. 4	Score 2 vs. 3	Score 2 vs. 4	Score 3 vs. 4
DLD system	0.004*	0.079	0.901	0.488	0.189	0.011*	0.594	0.034*	0.107
Thoracic radiologists	0.579	0.998	0.687	0.405	< 0.001*	0.001*	< 0.001*	0.007*	0.536
Nonthoracic radiologists	0.082	0.029*	0.473	0.672	0.050	0.001*	0.0120*	< 0.001*	0.028*
Radiology residents	0.266	< 0.001*	0.007*	0.240	0.006*	< 0.001*	0.030*	< 0.001*	0.004*

*DLD* Deep learning-based nodule detection, *LLF* Lesion localization fraction

^*^p < 0.05 was regarded as statistically significant

### Diagnostic performance of the observers according to the nodule location: without vs. with the DLD system

**Fig. 4:**
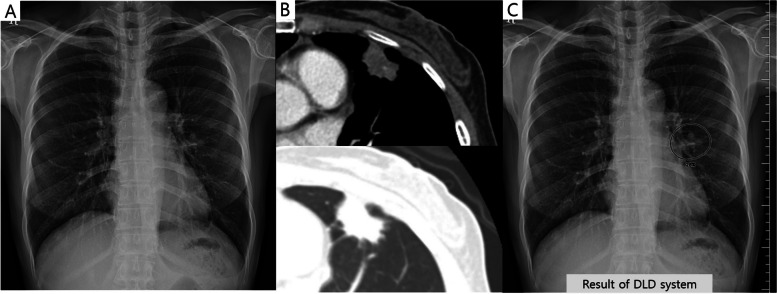
A case of a 50-year-old female patient with a pulmonary nodule. The nodule is located in the left paramediastinal area (danger zone) on the chest radiograph (**a**). Chest computed tomography revealed a 2.5-cm irregular shape nodule in her left upper lobe (**b**). The deep learning-based nodule detection (DLD) system detected this nodule and suggested an abnormality probability of 28% (**c**). All thoracic radiologists (2/2) detected this nodule without the DLD system. However, among other observers, only one radiology resident detected this nodule (1/6) without the DLD system. Using the DLD system, all the observers were able to detect this nodule

**Fig. 5:**
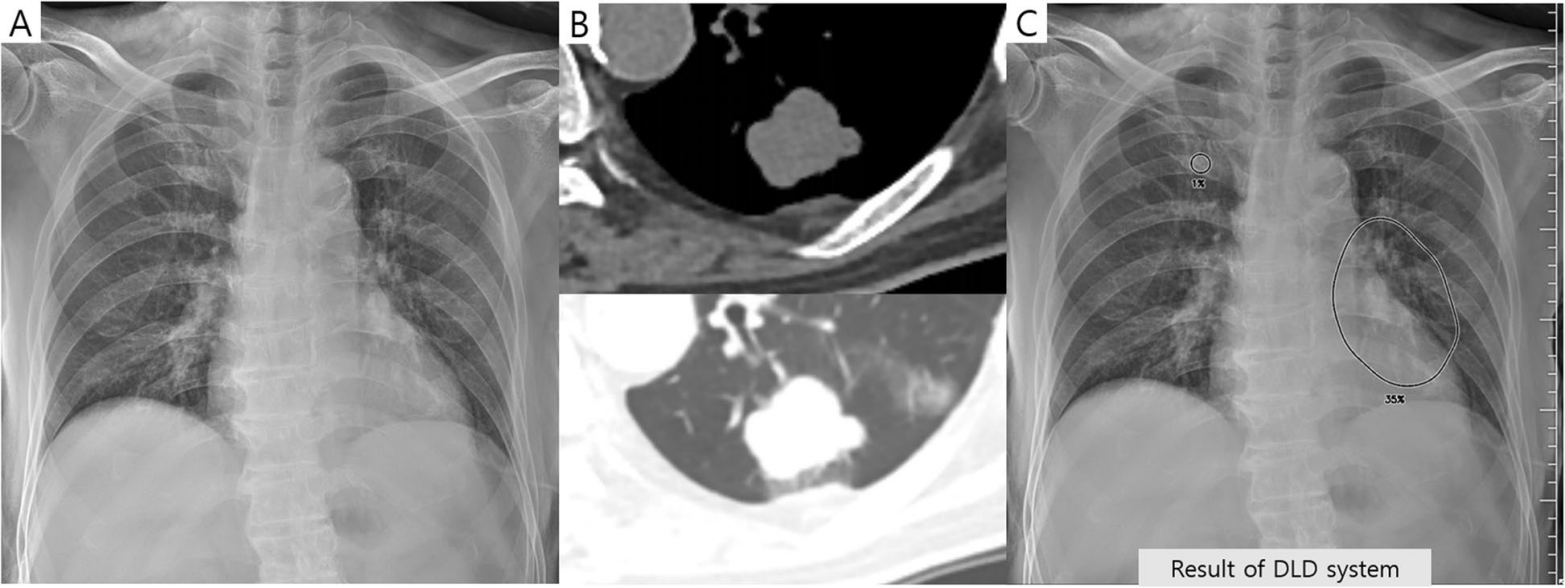
A case of an 85-year-old male patient with a pulmonary nodule. The nodule is located in the left paramediastinal area (danger zone) on the chest radiograph (**a**). Chest computed tomography revealed a 2.9-cm irregular lobulating contour nodule in his left lower lobe (**b**). The deep learning-based nodule detection (DLD) system detected this nodule and suggested an abnormality probability of 35% (**c**). One thoracic radiologist (1/2) and two non-thoracic radiologists (2/2) detected this nodule without the DLD system. Among radiology residents, only one radiology resident detected this nodule (1/4) without the DLD system. Using the DLD system, all the observers were able to detect this nodule

As aforementioned, the overall diagnostic performance of TRs and RRs was significantly improved when referring to the results of the DLD system. However, analysis of the diagnostic performance according to the nodule location revealed that only the diagnostic performance of RRs significantly improved with the DLD system for the detection of PNs in the DZ, from 56.7 to 65.6 (*p *= 0.021) (Table 6, Figs. 4 and 5). For the detection of nodules in the NDZ, no significant difference in the diagnostic performance of the observers was observed between the reading sessions.

**Table 6:** Comparison of the observers’ LLF according to nodule location without versus with the DLD system

	Session 1	Session 2	*p-*value
Danger zone nodules			
Thoracic radiologists	81.7 (178/218)	81.7 (178/218)	> 0.999
Non-thoracic radiologists	56.4 (123/218)	61.9 (135/218)	0.735
Radiology residents	56.7 (247/436)	65.6 (286/436)	0.021*
Non-danger zone nodules			
Thoracic radiologists	84.6 (181/214)	86.9 (186/214)	> 0.999
Nonthoracic radiologists	77.1 (165/214)	79.9 (171/214)	> 0.999
Radiology residents	75.5 (323/428)	74.8 (210/428)	0.462

*DLD* Deep learning-based nodule detection, *LLF* Lesion localization fraction

^*^p < 0.05 was regarded as statistically significant. For group-averaged comparison, corrected *p-*values are presented (multiplied by 3). Denominator for the LLF calculation corresponds to the total number of true lesions multiplied by the number of readers in the respective group

## Discussion

In our study, we demonstrated that the performance of the DLD system was poorer in detecting PNs in DZ than in NDZ (LLF, 64.2 vs. 83.2;* p *= 0.008), even though the nodules in the DZ were significantly larger than those in the NDZ. This result is similar to that obtained by non-expert human observers. Nevertheless, the performance of detecting DZ nodules by non-expert radiologists improved (56.4 to 61.9 in NTRs, *p *= 0.735; 56.7 to 65.6 in RRs, *p *= 0.021) when the DLD system was used.

Since deep learning-based research is being actively investigated in the field of radiology, many artificial intelligence-based products have been developed and commercially used [16]. In actual clinical practice, these artificial intelligence products for chest radiographs are being used to assist radiologists or clinicians; thus, artificial intelligence as an assistant would be expected to help radiologists or clinicians not miss critical abnormalities and assure their interpretation [17]. Particularly, the detection of nodules could be enhanced with the use of artificial intelligence-based products since their detection in the DZ of chest radiographs is a challenging task for radiologists. Therefore, one of the expected roles of artificial intelligence-based products is to assist radiologists in not missing nodules in the DZ. We hypothesized that the performance of the DLD system would reach or even surpass the performance of human observers in the detection of PNs regardless of the location, since the DLD system was built using a large amount of data (15,809 CXRs) [14]. However, our study demonstrated that the DLD system showed a poorer performance in detecting PNs in the DZ than in the NDZ. This seems to be attributed to the nature of a deep learning-based algorithm that inevitably has a human perspective since it was trained and tuned based on CXRs provided by human radiologists.

For nodules in the DZ, the performance of the DLD system was lower than that of TRs (LLF, 64.2 vs. 81.7, *p *< 0.001). TRs with more experience in CXRs than NTRs and RRs showed similar nodule detection performance regardless of the nodule location (LLF, 84.6 in NDZ vs 81.7 in DZ;* p *= > 0.999). Therefore, we could expect an improvement in the performance of the DLD system if the DLD system was developed using a PN dataset considering the nodule location and a combined dataset of the posteroanterior and lateral views of CXRs.

Despite the DLD system showing poorer performance in the detection of nodules in the DZ, the ability of RRs to detect nodules in the DZ was improved when referring to the results of the DLD system (LLF, 56.7 vs. 65.6; *p = *0.021*)*. This finding suggests that the DLD system could help less experienced physicians detect nodules in the DZ. Thus, the DLD system could be useful in small community hospitals or healthcare screening institutions where experienced TRs may not be available.

Remarkably, the use of the DLD system resulted in a significant reduction in FPPI across all observer groups. The results were similar to those of a previous study conducted by another institution using the same DLD system [12]. When observers read CXRs, instead of considering the DLD system’s falsely detected lesions as true lesions, readers tended to interpret ambiguous lesions, whether they were nodules or not, as negative when the DLD system did not detect them. Consequently, the usage of the DLD system enhanced the readers’ confidence in affirming that the ambiguous lesion under consideration was indeed not a nodule.

We performed subgroup analysis to confirm whether the nodule location, size, and visibility affected the detection rate among nodules in the DZ. Notably, the DLD system detected nodules in the apical zone better than other nodules among the nodules in the DZ (LLF, 87.9 for apical nodules, 52.5 for paramediastinal nodules, 58.8 for retrodiaphragmatic nodules, respectively) and demonstrated a higher detection rate than other human observers in the detection of nodules in the apical zone (LLF, 87.9 for the DLD system, 84.9 for TRs, 69.7 for NTRs, 65.9 for RRs, respectively). This is probably attributed to the characteristics of the DLD system developed using the dataset of the tuberculous endemic area where PNs in the apical zones are easily observed. In addition, the DLD system showed better detection performance as the size and visibility scores increased. These results are similar to those obtained by human observers.

Our study has several limitations. First, we only used CXRs obtained from one institution. However, since the DLD system that we used was developed from image datasets from multiple centers and more than 15 different global vendor X-ray machines [14], this disadvantage could be compensated to some extent. Second, since the DZ and NDZ nodules were selected with a 1:1 ratio and were included in the dataset, the distribution could vary from the ratio in the real world. Therefore, the overall performance of the DLD system and observers could be underestimated. Third, CXRs of the lateral view were not evaluated. However, to the best of our knowledge, no commercially available DLD system capable of evaluating lateral view CXRs exist. Thus, developing and studying deep learning algorithms trained using lateral view radiographs are warranted. Fourth, since our study focused on per-nodule detection according to the location of PNs, per-image specificity was not evaluated.

## Conclusion

The nodule detection performance of the DLD system was lower for detecting PNs in the DZ. Therefore, improving the detection performance of the DLD system through training DZ nodules of more data is preferred. Also, a lot of clinical practice with CXRs can improve observers’ performance of nodule detection. Nonetheless, because the DLD system has the clinical value of aiding less experienced observers to detect PNs in the DZ, it can be useful in circumstances without experienced radiologists.

### Supplementary Information

**Additional file 1:** **Table S1.** Information of the manufacturers and techniques of the CXRs.

## Data Availability

The datasets analyzed during the current study are not publicly available owing to institutional policy but are available from the corresponding author on reasonable request.

## Abbreviations

CT: Computed tomography
CXR: Chest radiographs
DLD: Deep learning-based nodule detection
DZ: Danger zone
FPPI: False-positive findings per image
JAFROC: Jackknife alternative free-response receiver operating characteristic
LLF: Lesion localization fraction
NDZ: Non-danger zone
NTR: Non-thoracic radiologist
PN: Pulmonary nodule
RR: Radiology resident
TR: Thoracic radiologist

## References

[1] Kligerman S, Cai L, White CS (2013). The effect of computer-aided detection on radiologist performance in the detection of lung cancers previously missed on a chest radiograph. J Thorac Imaging.

[2] Nam JG, Park S, Hwang EJ (2019). Development and validation of deep learning-based automatic detection algorithm for malignant pulmonary nodules on chest radiographs. Radiology.

[3] de Hoop B, De Boo DW, Gietema HA (2010). Computer-aided detection of lung cancer on chest radiographs: effect on observer performance. Radiology.

[4] Lee KH, Goo JM, Park CM, Lee HJ, Jin KN (2012). Computer-aided detection of malignant lung nodules on chest radiographs: effect on observers' performance. Korean J Radiol.

[5] Quekel LG, Kessels AG, Goei R, van Engelshoven JM (1999). Miss rate of lung cancer on the chest radiograph in clinical practice. Chest.

[6] Shah PK, Austin JH, White CS (2003). Missed non-small cell lung cancer: radiographic findings of potentially resectable lesions evident only in retrospect. Radiology.

[7] Muhm JR, Miller WE, Fontana RS, Sanderson DR, Uhlenhopp MA (1983). Lung cancer detected during a screening program using four-month chest radiographs. Radiology.

[8] Monnier-Cholley L, Arrive L, Porcel A (2001). Characteristics of missed lung cancer on chest radiographs: a French experience. Eur Radiol.

[9] de Groot PM, Carter BW, Abbott GF, Wu CC (2015). Pitfalls in chest radiographic interpretation: blind spots. Semin Roentgenol.

[10] Goun Choi BDN, Hwang Jung Hwa, Kim Ki-Up, Kim Hyun Jo, Kim Dong Won (2020). Missed lung cancers on chest radiograph: an illustrative review of common blind spots on chest radiograph with emphasis on various radiologic presentations of lung cancers. J Korean Radiol Soc.

[11] Jang S, Song H, Shin YJ (2020). Deep learning-based automatic detection algorithm for reducing overlooked lung cancers on chest radiographs. Radiology.

[12] Sung J, Park S, Lee SM (2021). Added value of deep learning-based detection system for multiple major findings on chest radiographs: a randomized crossover study. Radiology.

[13] Kim EY, Bista AB, Kim T (2017). The advantage of digital tomosynthesis for pulmonary nodule detection concerning influence of nodule location and size: a phantom study. Clin Radiol.

[14] Park S, Lee SM, Lee KH (2020). Deep learning-based detection system for multiclass lesions on chest radiographs: comparison with observer readings. Eur Radiol.

[15] Chakraborty DP (2005). Recent advances in observer performance methodology: jackknife free-response ROC (JAFROC). Radiat Prot Dosimetry.

[16] van Leeuwen KG, Schalekamp S, Rutten M, van Ginneken B, de Rooij M (2021). Artificial intelligence in radiology: 100 commercially available products and their scientific evidence. Eur Radiol.

[17] Hwang EJ, Goo JM, Yoon SH (2021). Use of artificial intelligence-based software as medical devices for chest radiography: a position paper from the Korean Society of Thoracic Radiology. Korean J Radiol.

