# Supplementary material for: The diagnostic performance and clinical value of deep learning-based nodule detection system concerning influence of location of pulmonary nodule
Source: Insights Imaging. 2023 Sep 19;14:149. doi: 10.1186/s13244-023-01497-4 (PMC10509107; doi:10.1186/s13244-023-01497-4)
Supplement: Supplementary file 1 — Additional file 1: Table S1. Information of the manufacturers and techniques of the CXRs. [file 13244_2023_1497_MOESM1_ESM.docx]

**The Diagnostic Performance and Clinical Value of Deep Learning-based Nodule Detection System Concerning Influence of Location of Pulmonary Nodule**

**ELECTRONIC SUPPLEMENTARY MATERIAL**

**Table S1.**

Information of the manufacturers and techniques of the CXRs

| **Manufacturer** | | **Model name** | **Technique** |
| --- | --- | --- | --- |
| Carestream | USA | DRX-Revolution | Digital radiography |
| DongKang | South Korea | INNOVISION | Digital radiography |
| GE Healthcare | USA | Discover XR 650 | Digital radiography |
| Listem | South Korea | DRS | Digital radiography |
| Philips Medical systems | Netherlands | DigitalDiagnost | Digital radiography |
